# Hospital and Institutional News

**Published:** 1918-03-16

**Authors:** 


### HOSPITAL AND INSTITUTIONAL NEWS.

### A FINE SPEECH.

THERE is a newspaper convention that soldiers cannot make speeches, a convention, we fear, grounded more on Tennyson's phrase, "Theirs not to reason why," than on observance of the facts. At all events Sir William Robertson made a fine speech the other day at Lincoln on visiting the 4th Northern General Hospital. After remarking on the achievement of medicine in preventing the occurrence of "a single epidemic" in even the notoriously unhealthy theatres of the war, he went on to say that the incapacitated men must be provided for. "Let me add," he went on, "that the gratitude which begins and ends in words is not of much use. The time comes when it must take a practical form—the form of hard cash." People at home were making great sacrifices. They might be asked to submit to more before the war could be brought to a conclusion which would remove a disgrace from civilisation. He defined this disgrace as "the waste of life, the waste of labour involved in nations maintaining great armies for the purpose of destroying each other." This criticism of a great soldier of the crime of militarism is very striking. We have only to remember that Prussia is not a nation but a creed, to endure to the end, to pursue it to utter destruction, and to avoid fogging that plain issue by refusing to waste time discussing terms of peace. Such discussion means negotiation, and negotiation means that Prussia is to escape the penalty of that defeat for which we are determined to fight, since anything short of that becomes merely a phrase of loose thought whose meaning is the escape of a criminal.

### "THE CROWNING ACT OF HINDRANCE."

THE scheme for a medical school at Cardiff in connection with the South Wales University College has met with so much official discouragement that, we suppose, the story of the fight of a few men of public spirit like Sir W. J. Thomas and Colonel Bruce-Vaughan would not be complete unless there were to be "a crowning act of hindrance." In these words the *South Wales Daily News* describes with natural indignation the proposal to commandeer the first section of the new building, now nearly complete, for the latest of our rich crop of Government Departments. The journal remarks that the existence of "a private proposal and special effort to divert the building from the purpose of a medical school is common knowledge." Well, every ambitious scheme has local enemies jealous of its aims and grudging them success. Only lately the fatuous proposal to commandeer the British Museum was barely squelched by an unflinching protest. The name of the author of that absurdity should have been published, for he was probably one of our commercial adventurers, who mistake wealth for culture, and the astuteness of the tradesman for statesmanship. But it was wanted—was it not?—for the Air Board! If the British Museum, perhaps the one disinterested wellspring of wisdom that we have, but narrowly escaped, it is hardly to be supposed that Cardiff's new medical school would not be pounced on. We hope by now that this attempt has also been scotched. The remedy for these absurdities is, *after they have been defeated*, to publish the name of their author and that of the head of the Department which it was suggested should have occupied the building. If such an one happens to be a newspaper proprietor a fit of modesty, quite suddenly, enshrouds his head. But he must not be allowed to make a convenience of the virtues in this way. Cannot the chief of the Welsh nation, Mr. Lloyd George the indefatigable, stretch forth his hand? Then this greatly needed Medical School and University College for Wales will be freed from its shameful assailants without further bother or delay.

### THE PRUDENTIAL ASSURANCE COMPANY.

SIR THOMAS DEWEY, the very able and trusted chairman of this great company, the number of whose policies, including annuities, in force at the end of 1917 was 934,075, had a wonderful statement to make to the shareholders at the sixty-ninth annual meeting on the 7th instant. A full summary of the report will be found on another page, and we commend its perusal to those of our readers who are interested in insurance, or who have a love for figures and follow with pleasure the progressive development and results achieved by great commercial enterprises. They will see that it is quite impossible to deal with the salient features of this report within the space at our disposal, nor is this necessary, for the report itself is so full of interest, so tersely and clearly put, and so remarkable in the facts it contains as to give it a convincing and cumulative force which all who read must feel. Sir Thomas Dewey in his speech insisted that the Prudential had taken no inconsiderable part in matters relative to the war, both as regards the shouldering of war burdens, and affording active support to the Government in every possible way. The Prudential has consistently used every endeavour to help forward the War Savings movement. Its actuary, Mr. Burn, has been a member of the Central Committee from the first, and has spent a large portion of his time in active work of an important nature. Its statistical and actuarial departments have undertaken, and are still undertaking, free of all cost to the Government, an amount of work in connection with the accounts of over 40,000 War Savings Associations throughout the country, which Sir Thomas Dewey believes would astonish some Government departments. The company's own War Savings Association in the head office is the largest in existence, and many of the Prudential's skilled staff in accountancy are voluntarily giving their spare evenings to the work of auditing the accounts of other War Savings Associations. This patriotic service in regard to national objects has in no way interfered with the carrying on of the Prudential's business with a degree of success which is probably unique. The total income of the year was £19,880,500, an increase of £1,179,366 over the previous year. The increase in the premiums received was £754,371. The new business in the ordinary branch showed a record increase. The new premium income, £567,472, was much in excess of that of the previous year.

### SIR THOMAS DEWEY.

There are many other points of importance and interest which will be found set forth in full on pages 8 and 9 of the *Westminster Gazette* of the 8th instant, where a verbatim report of Sir Thomas Dewey's speech is given. It is probably one of the most remarkable speeches ever made at a meeting of a great insurance company, and we congratulate Sir Thomas Dewey on the striking results which he clearly brought out in the course of his speech of great power and general interest. Sir Thomas Dewey has done so much for nurses as deputy-chairman of the Royal National Pension Fund, and has rendered much public service of supreme value in other directions, that readers of The Hospital have long held him in high esteem as a man of the day.

### PATIENTS' FEES OR DISTRICT CONTRIBUTIONS.

Like other hospitals, Southwold Cottage Hospital finds itself compelled to realise capital because the districts which rely on it for treatment do not rely on themselves to provide it with an income. When it was lately proposed that no cases, save accidents, should be admitted from districts which provided no financial assistance, Dr. Mullock suggested that a better way would be to charge an adequate fee. Hitherto, it is asserted, the fees charged have not even covered the cost of food, so there is clearly room for some revision. A committee has therefore been appointed to decide on a fee which shall secure the hospital against loss. We hope that matters will not stop there. It is a mistake to refuse to admit patients for whom there is room and who are otherwise eligible. The proper plan is to bring home to each district what it demands of the hospital in service, and to compare this with that which it gives. The point can be pressed, since some districts are better organised or more public-spirited than others. With persistence a series of district funds can be arranged, which will be encouraged by the regular publication of a table of what their patients cost and what the district gives. In this way support can be secured. For a voluntary hospital which relies for its support upon the sale of capital and fees from patients will quickly alienate all regard and public interest from it.

### FROM THE POINTS OF THE COMPASS.

The Great Northern Central Hospital is well remembered—even in far distant places—by those who have once been inside its wards. Take these instances: In spite of the submarine, recent overseas mails have brought to the hospital an annual subscription from a lady who is now at an American University; from St. Vincent, Cape Verde, a former hospital sister sent £3 3s., the lady's second contribution to the Nurses' Home Fund, while an officer serving with the British Forces in Portuguese East Africa has forwarded his cheque for £10, with a pencilled letter, saying it was "a small expression of appreciation for the excellent way I was treated when in the care of your hospital." Overseas mails in these days have a peculiar fragrance when they come, and this recognition of persistent affection for the hospital is the best tribute which its work could have.

### DRY-ROT IN THE BUILDING.

Mr. H. D. Acland has publicly drawn attention to what he describes as a dangerous condition in the fabric of Falmouth Cottage Hospital. Their buildings, he said, had turned sick. That was most dangerous in a hospital, and without being an alarmist he wished to remind the hospital's supporters that dry-rot in certain conditions caused periodic and peculiar diseases in buildings. Such a condition should not be allowed to exist a day after it had become known. He hopes that funds will be immediately raised to meet the cost of curing the condition. We hope so, too. The publication of an architect's report explaining the meaning of dry-rot and the dangers which it occasions should arouse the people of Falmouth and increase not merely their general information, but their personal interest in the Cottage Hospital itself.

### HOMŒOPATHIC HOSPITALS AND THE COLLEGE OF NURSING.

The Hahnemann Hospital, Liverpool, has raised a protest against the reduced grant received from the Hospital Sunday and Saturday Fund. It bases this on the value of its own work, and on the fact that the Fund's Committee was able to distribute a larger sum than ever last year. Speaking on the former aspect of the question, Mr. J. Carlton Stitt said they were proud of the success of the nurses they had trained, but if the College of Nursing Bill became law the Council of the College would be empowered not to recognise the hospital as a training-school because of the relatively small number of beds. Homœopathic hospitals are not often large and their future in this regard merits consideration.

### A MATERNITY HOSPITAL'S GOOD FORTUNE.

The annual meeting of the Leicester Maternity Hospital, presided over by Sir Thomas Cope, Bart., was marked by the excellence of the speeches and by a generous gift of £1,000 in memory of the late Mrs. T. Fielding Johnson, whose work for the charitable institutions of Leicester formed the subject of a biographical notice in The Hospital of November 17 last. In moving the adoption of the annual report Sir Thomas Cope referred to the great loss the hospital had sustained in the deaths of two of its founders, Sir Edward Wood and Mrs. Fielding Johnson. The Council felt that there should be a memorial to Mrs. Johnson which should serve to record her close association with the hospital, and they were, therefore, very greatly indebted to Mrs. Wallace Bruce, her daughter, for the gift of £1,000 for the endowment of a ward to be called the "Agnes Johnson" ward. The Leicester Town

Council had also made a grant to the hospital of £100 in return for the use of twenty-five beds. The Mayor of Leicester, in seconding, commended the work of the hospital to the residents, and considered that there was a great deal of encouragement to be found in the report presented. Commendatory speeches were also made by Mrs. Wood, wife of the Bishop of the diocese; the Rev. F. R. C. Payne, prison chaplain; and Dr. Millard, the medical officer of health.

### THE VICAR OF BRIGHTON'S DICTUM.

The month of February brought with it the usual crop of annual meetings of the medical charities of Brighton and Hove. A feature of the meetings this year, recalling the work of 1917, was the tribute which the new Vicar of Brighton was able to pay to the various institutions. Canon Dormer Pierce has not yet been in the town twelve months, but he has taken the opportunity of personally visiting all the local medical charities, and he has been so impressed with the work that he readily acceded to the demand upon him to be chairman of the annual meeting of more than one institution and a speaker at others. At one he said, "This is the first time in twenty-five years' experience as a parish priest in very poor parishes that I have found this work among women and children adequately done." Canon Dormer Pierce's first-hand experience of the excellent manner in which the medical work is organised at Brighton should make a direct appeal to those who come to Brighton and Hove, not only for health, but to escape air-raids. Both towns are full of them.

### A RASH M.P.

Sir Edgar Jones, M.P., speaking at a musical performance at Forndale in aid of Netley Hospital, indulged in the following comparison. Alluding to his recent visits to France, he said that the wounded were having much more tender-hearted consideration in France than in Great Britain. The military hospitals in this country, he added, were better than the civilian hospitals before the war. It would be interesting to know on what observations Sir Edgar Jones bases these assertions, and what occurrences prompted him to make this strange comparison.

### A HOSPITAL ROCK-GARDEN.

The war has encouraged a number of new treatments, educational and therapeutic, which have been widely adopted, especially in relation to orthopædic cases. To these must be added the rock-garden which is a peculiarity of the Prince of Wales' Hospital for Limbless Soldiers and Sailors at Cardiff. In our last issue we gave an account of the Prince of Wales' visit when he opened this hospital on February 20. But the rock-garden attached to the hospital deserves separate mention. Not only do men have to be trained to use their artificial limbs, but these, too, have to be tested by the wearer, for both man and limb have to learn to accommodate themselves to variations of gradient and camber which pass unnoticed by the man who is "sound on his pins." To use an artificial limb on a gradient is a skill which cannot be acquired by walking with it on the flat. For this reason a walking-ground of various levels has been designed at the Prince of Wales' Hospital and is called locally "Wild Wales." Its rough stone paths have levels, ascents, and declivities. The up-gradients vary to the limit of 1 in 3½. The camber of the paths also varies. Instead of being semicircular as in the ordinary path, with more or less flatness at the crest, in one stretch the path is laid so that the right foot is higher than the left, and in another the left is higher than the right. The armless men also find opportunities for educational treatment in the garden under the guidance of Mr. T. Williams, who, to meet his own need after a mishap as a collier, invented twenty odd years ago an ingenious working arm. The rock-garden possesses pits, where loading sand and gravel can be practised together with the allied work of wheeling barrows and using picks and mattocks. An illustrated handbook has been published by the hospital authorities, in which the whole scheme, including the hospital's origin and development, has been described in detail. Inquiries concerning it should be addressed to the secretary of the hospital, Mr. G. D. Shepherd.

### RETURN OF SECRETARY FROM THE FRONT.

Major E. A. Attwood, late R.F.A., has now returned to the London Homœopathic Hospital to resume his duties as secretary, having, for reasons of health, been retired from the Army on half-pay. Major Attwood, on March 8, delivered a lecture on his two years' experiences in France to the members of the Hospital Officers' Association at their headquarters in Bedford Square. The lecture was illustrated by views from the most refractory magic-lantern we have had the misfortune to meet. This inconvenience, however, was amply made up for by the fund of varied information and anecdote supplied by the lecturer. There was, however, one slide which was projected on the screen with much clearness. It represented a French and English soldier conversing by the side of a railway line. At this point of the lecture a highly respected veteran London secretary rose and said: "If I may interrupt the lecturer, may I ask Major Attwood if he knows who is the English soldier before us?" The lecturer was not aware of the identity of the soldier, and the secretary who had intervened said, "It may interest the audience to know that he is my son, who, I may add, is no longer on this planet." The pathos of this extraordinary coincidence was apparent to all, and the announcement was received with a murmur of profound sympathy.

### MEDICAL "CLIQUISM" IN DUDLEY.

The appointment of honorary surgeons, even in the case of big institutions, is not usually a matter of public interest, but the proverbial exception to this rule has occurred in connection with the Guest Hospital, Dudley. The election committee met on March 8 to appoint an honorary surgeon, and was reminded in advance that applications of highly qualified candidates would be welcomed, whether, it was added, they happened to be resident in Dudley or outside. Critics remarked on the "cliquism" of Dudley and on its habit of refusing to entertain applications from medical men who happened to live technically outside the sacred area, even if living only twenty minutes away and possessed of the stock mechanical aids, a motor-car and a telephone. It was evidently feared that the election committee would be hampered by an extraneous and a native difficulty, the one a list of applications restricted to a handful of Dudley doctors, the other a want of courage to break through the alleged tradition of the clique. If there is this want of public confidence it is well that it should be expressed, and, as in this case, put forward quite clearly before the election occurred. The timid critic chooses to complain when he is too late to be listened to or respected.

### MR. HAYES FISHER ON LABORATORIES.

At the recent annual meeting of the County Councils Association Mr. Hayes Fisher, President of the Local Government Board, remarked that though only two years had passed since the report of the Commission on Venereal Diseases, fifty-seven out of sixty-two County Councils had adopted schemes on the lines suggested. The County Councils, indeed, had proved so willing to carry out a great policy of public health that they would not be surprised if the Government requested them to branch out into other fields of public service. The value of laboratories and their aids to scientific diagnosis had been proved in all serious attempts at the prevention of disease. In that field, added Mr. Hayes Fisher, County Councils might be asked to occupy an important position.

### THE "SEARCHLIGHT" AND THE FOOD PROBLEM.

There is generally at least one piece of interesting criticism in the *Searchlight*, the monthly gazette of the 2nd Western Hospital, Manchester. The February number is no exception to this rule, as will be clear from the following quotation: "We agree that the Home Forces should share with the civilian population whatever privations are necessitated. . . . We also accept the assumption that men engaged on clerical and other work of a sedentary nature do not require as much animal food as men engaged in rougher kinds of manual labour. There are, however, one or two facts which those in authority should be acquainted with. In the first place, it is a mistake to assume that there are any large number of men in the R.A.M.C. engaged in clerical work. Most of the clerical work is now done by women. The male staff, except in the case of a few heads of departments, are engaged on work quite as laborious as that done by the armies in the field. What is called the General Duty Section is the largest section in the Home Units at the present time. Another delusion . . . is that the R.A.M.C. staff get the same quality of meat as the patients. A short spell in the steward's store of any large hospital would bring speedy disillusionment. Eight ounces of staff meat, including bone and gristle, would try the teeth and the digestion of even a Food Controller, without doing much for his appetite. If it were necessary to reduce the meat ration, in the name of all that is merciful why did he not begin with the bone!" We only wish that other gazettes were capable of such criticism. The sharpness of the edge gives some idea of the weight of knowledge behind it.

### "MUDHOOK": A DIVISIONAL JOURNAL

The Journal of the 63rd (R.N.) Division, known as the *Mudhook*, a copy of which has just reached us, is believed to have created a record for a B.E.F. publication in the figures inscribed on the January issue, "Vol. I., No. 3." The paper seems as tenacious as the Division of its life. That it is not wholly a whimsey may be judged from the fact that the original editorial reached the Censor only, and for the half-franc which it costs the men of the battlefields and rest-camps where it circulates have some very vivid writing to read. Lieut. A. P. Herbert deserves special mention for his verse. Even *Punch* has found him out. But the verses reprinted from that periodical have not the acid bite and even recurrent rhythm which reflects so accurately the wearisome monotony of war in his admirable stanzas "To the Best Battalion." There are some witty proverbs known as "Perverted Sayings," of which the best are "Brevity is the soul of kit," and "A clear sky at night is the Gotha's delight, and a barrage in the morning is Fritz's warning." The *Mudhook* is extra-illustrated by an etching of Gavrelle, whose etcher, alas! has cleverly concealed his signature among the grass which fills the foreground of this bare landscape of stripped and scattered tree-stumps. Elsewhere we find his name, Mr. Wilson. The motto suggested for the Jewish Battalion is "No advance without security." Acidity is wit, and seasons observation. There is, too, a skit entitled "Conducted Tours in France," and even the advertisements are jokes.

### THIS WEEK'S DRUG MARKET.

Once more practically all the price changes of the week are in an upward direction. A further advance of one shilling per ounce in the price of strychnine is announced by the makers; the advance is due to the high cost of nux vomica and the shortage of labour. The Food Controller having forbidden the use of lard for mercury ointment, makers are not offering this preparation until a new base is decided upon. The advanced price of bromides is firmly maintained. The scarcity of sugar of milk has become still more acute, and the price is now quoted by the pound as often as by the hundredweight. In spite of the arrival of a considerable supply of saccharin there is no decline in the exorbitant price; as yet no decisive action has been taken by the Food Controller. Japanese dementholised peppermint oil is dearer, as also is menthol. Quinine is unchanged in price, in spite of the much reduced stocks. Honey is again dearer. Other articles which are advancing in price are cream of tartar, cascara sagrada, cassia oil, citronella oil, artificial oil of wintergreen, and coumarin. It is becoming more and more difficult to transact business in various medicinal products, and makers are not infrequently obliged to put their customers on rations.

